# Methylene Blue Inhibits Caspases by Oxidation of the Catalytic Cysteine

**DOI:** 10.1038/srep13730

**Published:** 2015-09-24

**Authors:** Prateep Pakavathkumar, Gyanesh Sharma, Vikas Kaushal, Bénédicte Foveau, Andrea C. LeBlanc

**Affiliations:** 1 Bloomfield Center for Research in Aging, Lady Davis Institute for Medical Research, Jewish General Hospital, Montreal, Quebec, Canada; 2 Department of Neurology and Neurosurgery, McGill University, Montreal, Quebec, Canada

## Abstract

Methylene blue, currently in phase 3 clinical trials against Alzheimer Disease, disaggregates the Tau protein of neurofibrillary tangles by oxidizing specific cysteine residues. Here, we investigated if methylene blue can inhibit caspases via the oxidation of their active site cysteine. Methylene blue, and derivatives, azure A and azure B competitively inhibited recombinant Caspase-6 (Casp6), and inhibited Casp6 activity in transfected human colon carcinoma cells and in serum-deprived primary human neuron cultures. Methylene blue also inhibited recombinant Casp1 and Casp3. Furthermore, methylene blue inhibited Casp3 activity in an acute mouse model of liver toxicity. Mass spectrometry confirmed methylene blue and azure B oxidation of the catalytic Cys163 cysteine of Casp6. Together, these results show a novel inhibitory mechanism of caspases via sulfenation of the active site cysteine. These results indicate that methylene blue or its derivatives could (1) have an additional effect against Alzheimer Disease by inhibiting brain caspase activity, (2) be used as a drug to prevent caspase activation in other conditions, and (3) predispose chronically treated individuals to cancer via the inhibition of caspases.

Methylene blue, a tricyclic phenothiazine (also known as methylthionine hydrochloride), has a history of diverse medical applications stretching over a century for treatment of enzymopenic hereditary methemoglobinemia, acute acquired methemoglobinemia, urinary tract infections, malaria, septic shock, and hepatopulmonary syndrome^1^. Alzheimer disease (AD) is a progressive neurodegenerative disorder showing abundant deposits of β-amyloid peptide (Aβ) plaques, intracellular neurofibrillary tangles (NFTs) consisting of Tau protein, and the loss of synapses^2^. Presently, inhibitors of Tau aggregation are being considered as therapeutic interventions against AD, and methylene blue disaggregates Tau NFTs^3^. Methylene blue, and its demethylated derivatives azure A and azure B, were initially identified as blocking *in vitro* Tau-Tau aggregation identified as paired helical filaments by electron microscopy^3^. Methylene blue was also shown to prevent heparin-induced Tau filament formation^4^. More recently, several studies have demonstrated the ability of methylene blue to prevent Tau aggregation in transgenic mouse models expressing the P301L or P301S Tau mutations associated with the formation of NFTs in mice and in human disease. Treatment of the rTg4510 human P301L transgenic mouse with methylene blue improved behavior slightly in treated 3 month old mice, reduced brain total Tau and phospho-Tau, and increased neuronal survival^5^. However, treatments in 16 month old rTg4510 mice did not have any effect on Tau levels, neuronal survival or brain atrophy^6^. The preventative nature of methylene blue was also observed in Tau∆K280 and TauRDK transgenic mice where treatments started in 1.5 or 9 month old mice, but not in 15 month old mice, showed improved cognitive behavior and a decrease in pathological Tau at 18 months of age^7^. Similarly, methylene blue treatment of the JNPL3 human P301L or the P301S transgenic mice decreased Tau pathology in brains^8^,^9^. Reduced Tau aggregation was also observed in JNPL3 organotypic brain slices^10^. Furthermore, methylene blue improved behavioral deficits and Tau pathology in C. elegans and Drosophila models^11^,^12^. Accordingly, methylene blue is currently in phase III clinical trials in human AD patients in the hope of stopping the progression of cognitive deficits and dementia^13^.

Methylene blue has been shown to clear Tau pathology through increased autophagy in JNPL3 organotypic slices^10^. Molecularly, methylene blue, and its mono and di-N-demethylated forms, azure B and azure A, were shown to interact with and promote the oxidation of Tau cysteine residues, retaining Tau in a monomeric conformation, thus preventing formation of fibrils and their toxic precursors^14^,^15^. The identification of this mechanism prompted us to ask whether methylene blue may modulate the activity of caspases, a group of cysteinyl proteases involved in inflammation and cell death.

One of the effector caspases, Caspase-6 (Casp6), has been highly implicated in age-dependent cognitive decline and in sporadic and familial AD pathology^16^,^17^,^18^,^19^,^20^. Furthermore, the expression of a self-activated form of Casp6 in the hippocampal CA1 of mice induces age-dependent cognitive deficits in episodic and spatial memory^20^. While Casp6 in cells and neurons does not induce the expected effector caspase-mediated rapid cell death^21^,^22^, Casp6 cleaves a number of cytosolic neuronal cytoskeleton or cytoskeleton-associated proteins, including Tau and α-tubulin^23^. Casp6 is implicated in axonal degeneration of developing and injured neurons^24^, nerve growth factor deprived mouse sensory neurons^25^,^26^,^27^,^28^, and primary human CNS neurons transfected to over-express AD-associated mutant amyloid precursor proteins^29^. Because of our expertise with *in vitro* and cellular Casp6 activity analyses, we initially studied the effect of phenothiazines on Casp6 activity, and further extended the research to Casp1 and Casp3.

Similar to other caspases, Casp6 is translated as a zymogen, comprised of a short prodomain, a p20 subunit containing the catalytic cysteine (Cys163), a linker region, and a p10 subunit^30^. The zymogen is cleaved at three distinct sites to remove the prodomain and linker regions in order to obtain an active enzyme. Interestingly, Casp6 can self-activate by intramolecular cleavage of its C-terminal linker-processing site^31^. Once activated, Casp6 forms a covalent tetrahedral intermediate where the de-protonated sulfur of the catalytic Casp6 Cys163 launches a nucleophilic attack on the scissile carbonyl of the substrate to generate an acyl enzyme intermediate^32^. Therefore, the catalytic cysteine must be in a reduced state to efficiently cleave protein substrates.

Since methylene blue inhibits the activity of Tau protein through its pro-oxidant activity on cysteines, here, we tested if it can also inhibit the cysteinyl caspase proteases. Our results showed that methylene blue and its derivatives efficiently inhibited active caspases *in vitro*, in cells, and *in vivo* at concentrations allowing phenothiazine-mediated Tau disaggregation. These results indicate that methylene blue or its derivatives could (1) have an additional effect in AD by inhibiting caspases, (2) be used as a drug to prevent caspase activation in other degenerative conditions, and (3) predispose chronically treated individuals to cancer via the inhibition of effector Casp3.

## Results

### Methylene blue and its derivatives inhibit the activity of caspases *in vitro*

**Figure 1:**
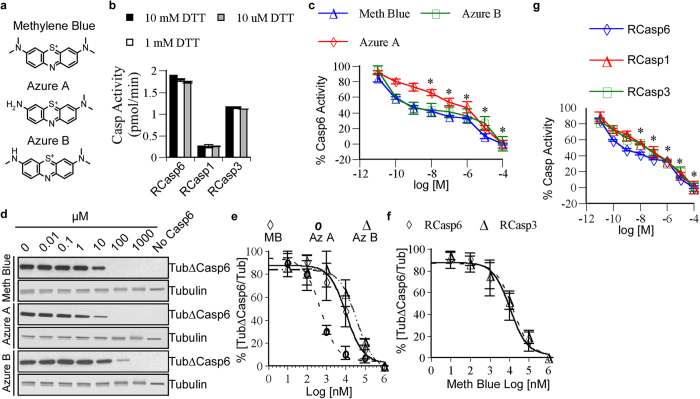
Inhibition of caspases *in vitro* by phenothiazines. (**a**) Chemical structures of methylene blue, azure A and azure B. (**b**) Activity of RCasp6, RCasp3, and RCasp1 with different dithiothreitol (DTT) concentrations. Data represent mean and SD of 3 independent experiments. (**c**) Dose-dependent inhibition of Casp6 in presence of methylene blue, azure A, and azure B. Each data point represents the mean and SD of 3 independent experiments. Statistical difference between no phenothiazines and addition of phenothiazines was determined by one-way ANOVA p < 0.0001 and post-hoc Bonferroni *p < 0.001. (**d**) Western blot analysis of the *in vitro* tubulin cleavage assay showing dose-dependent inhibition of Casp6 activity in the presence of methylene blue, azure A, and azure B. (**e**) Non-linear regression curve for dose-dependent inhibition of RCasp6 cleavage of tubulin in the presence of methylene blue and its derivatives. (**f**) Dose-dependent inhibition of RCasp6 and RCasp3 with methylene blue. For e and f, Data represent mean and SD of 3 independent experiments. (**g**) Dose-dependent inhibition of RCasp6, RCasp3, and RCasp1 with methylene blue. Data represent the mean and SD of 3 independent experiments. Statistical analyses conducted as in c.

To assess if methylene blue (Fig. 1a) can inhibit Casp6 activity through oxidation, the amount of dithiothreitol (DTT) required for measurable RCasp6, RCasp1, and RCasp3 activity was titrated on their preferred peptide substrate in an *in vitro* assay. Caspase activities were sufficiently retained with 10 μM DTT (Fig. 1b). Under these conditions, methylene blue and its mono- and di-demethylated derivatives, azure B and azure A (Fig. 1a), inhibited Casp6 activity in a dose dependent manner (Fig. 1c). Reduction in Casp6 activity was observed with only 100 pM methylene blue and azure B while 10 nM azure A was necessary for a significant inhibition. Because phenothiazines above the concentration of 100 μM exert a quenching effect on the caspase fluorogenic assays (see supplementary Fig. S1 online), the IC_50_ was calculated with an *in vitro* tubulin cleavage assay. The tubulin cleaved by Casp6 (Tub∆Casp6) was detected with a neoepitope antiserum whereas full length uncleaved tubulin (upper tubulin band) and Tub∆Casp6 (lower protein band) were detected with a full-length tubulin antibody by western blot analyses (Fig. 1d). The results show that methylene blue, azure A and azure B inhibit cleavage of tubulin by RCasp6 in a dose dependent manner (Fig. 1d). Quantitation of the Tub∆Casp6 relative to total tubulin yielded an IC_50_ of 10.6 μM (r^2^ = 0.87), 0.5 μM (r^2^ = 0.89), and 34.4 μM (r^2^ = 0.90) with methylene blue, azure A, and azure B, respectively (Fig. 1e). Methylene blue exhibited an IC_50_ of 14.2 μM on RCasp3-cleaved tubulin (Fig. 1f), confirming the dose dependent inhibition of RCasp6, RCasp3, and RCasp1 observed in the fluorogenic assay (Fig. 1g).

### Competitive mode of inhibition for phenothiazines on Caspase-6.

**Figure 2:**
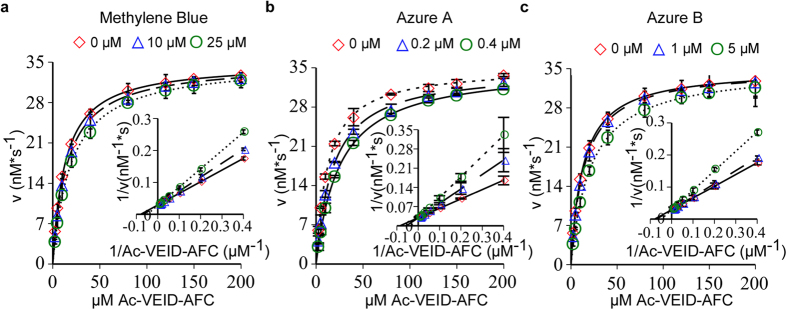
Michaelis-Menten kinetic analyses of phenothiazines on RCasp6. Initial enzyme velocity of Casp6 plotted against indicated concentrations of Ac-VEID-AFC substrate in presence of 0, 10 μM, and 25 μM methylene blue (**a**), 0, 0.2 μM, and 0.4 μM azure A (**b**), or 0, 1 μM, and 5 μM azure B (**c**). The inset represents the Lineweaver-Burk plot for methylene blue (**a**), azure A (**b**), and azure B (**c**) with Ac-VEID-AFC substrate showing competitive mode of inhibition. Data represent the mean ± S.D. of three independent experiments.

To determine the mechanism of methylene blue-mediated Casp6 inhibition, inhibitor kinetic studies were conducted with non-interfering concentrations of phenothiazine in the fluorogenic assay. Increasing concentrations of methylene blue (Fig. 2a), azure A (Fig. 2b) and azure B (Fig. 2c), resulted in increasing K_m_ values whereas the V_max_ values remained the same. A reciprocal (Lineweaver Burk) plot (inset) shows that the data intersect the y axis at the same point, indicating a competitive mode of inhibition. Fitting the data to the competitive inhibition model, and using nonlinear regression analysis, we determined the *K*_i_ values to be 50.5 μM ± 2.3 μM for methylene blue, 0.42 μM ± 0.02 for azure A, and 7.89 μM ± 0.2 μM for azure B, respectively. These results suggest that methylene blue, azure A, and azure B prevent binding of substrate in a competitive manner.

### Methylene blue and its derivatives inhibit the activity of caspases in cell lines and primary cultures of human neurons

**Figure 3:**
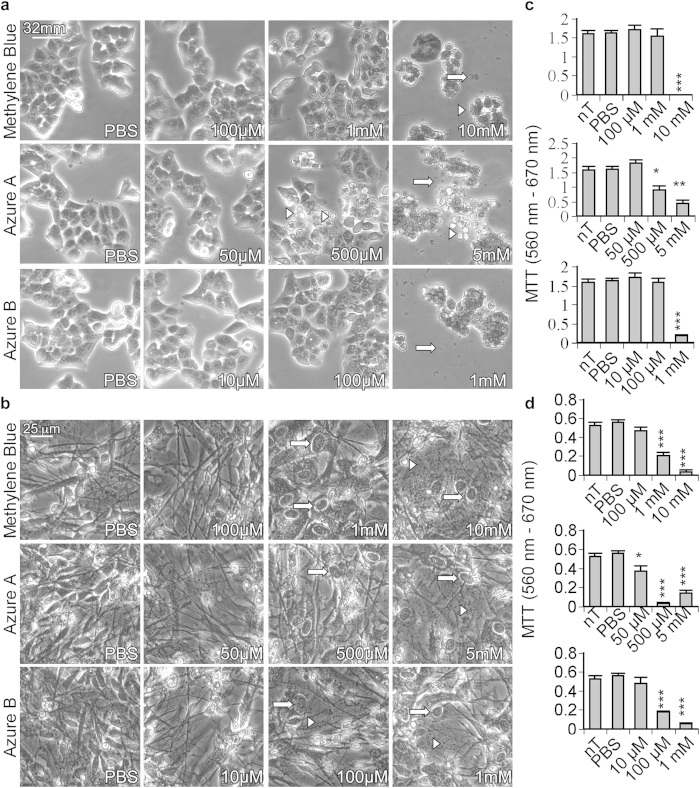
Toxicity of methylene blue, azure A, and azure B in HCT116 cells and human primary neurons. (**a**) Phase contrast images of (**a**) HCT116 cells and (**b**) human primary CNS neurons treated with an increasing concentration of methylene blue, azure A, or azure B for 2 hours. Round cells (white arrowheads) and cellular debris (white arrows) are indicated in (**a**), while fragmented axons (white arrowheads) and swollen cell bodies (white arrows) are shown for (**b**). The mitochondrial reductive potential of HCT116 cells and human primary neurons was measured by MTT assay in the presence or absence of methylene blue, azure A or azure B. HCT116 cells (**c**) or human primary neurons (**d**) were either non-treated (nT), treated with PBS, treated with 100 μM to 10 mM methylene blue, 50 μM to 50 mM azure A, or 10 μM to 1 mM azure B for 2 hours. Data represent the mean and SEM of 3 (**c**) or 4 (**d**) independent experiments. Statistical difference between phenothiazine treated samples and the PBS control was measured with a one-way ANOVA (p < 0.0001) and post hoc Dunnett’s analysis compared to PBS: *p < 0.05, **p < 0.01, ***p < 0.001.

Human colon carcinoma cells (HCT116) and human primary neurons were first observed by phase contrast microscopy after treatment with increasing concentrations of methylene blue and its derivatives (Fig. 3a,b). At 100 μM methylene blue, 50 μM azure A, and 100 μM azure B, HCT116 cells retained a morphology and confluence comparable to PBS-treated controls (Fig. 3a). Toxicity of high concentrations of phenothiazines was observed microscopically as rounded cell morphology, cell loss, and increased cellular debris in HCT116 cultures (Fig. 3a). Moreover, in human primary neurons, concentrations above 100 μM of methylene blue, 50 μM azure A, and 10 μM azure B increased cell body size and axonal fragmentation (Fig. 3b). The toxicity of phenothiazines was verified in MTT assays (Fig. 3c,d). Methylene blue treatment of HCT116 cells at concentrations of up to 1 mM had similar absorbance in the MTT assay as the PBS-treated cells, thereby indicating healthy mitochondrial function. However, 10 mM methylene blue decreased MTT absorbance to almost nothing, indicating toxicity. Azure A showed toxicity above 50 μM, while azure B was well tolerated up to 100 μM concentrations. Similar results were obtained on human primary neurons although the phenothiazines were more toxic on these cells than on HCT116 cells. The MTT signal represents reduced cell viability and not modulation of the oxidation-reduction state of MTT by phenothiazines because non-toxic concentrations of phenothiazines on HCT116 cells were observed to be toxic to human primary neurons in the MTT assay. Furthermore, the MTT results were consistent with the toxicity observed by microscopy.

**Figure 4:**
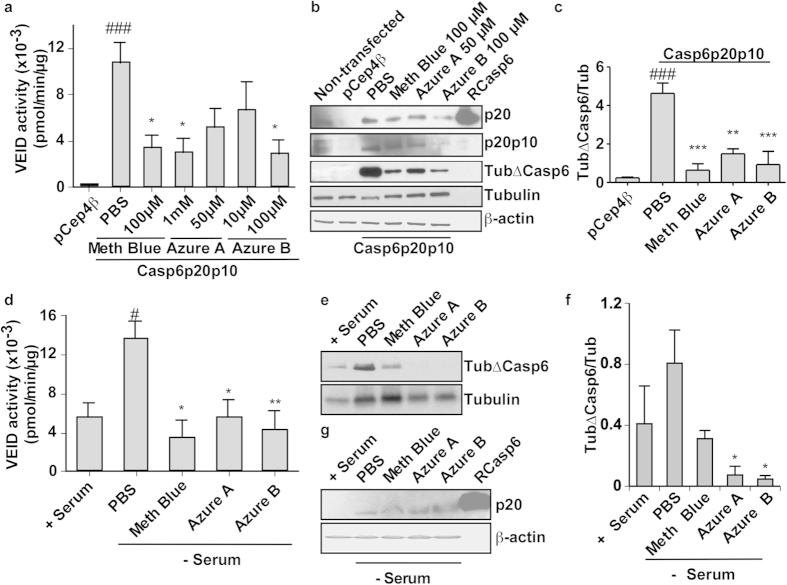
Methylene blue, azure A, and azure B inhibit active Casp6 in HCT116 cells and human primary neurons. (**a**) Casp6 VEIDase enzymatic activity in pCep4β vector- and pCep4βCasp6p20p10-transfected HCT116 cells treated with PBS, methylene blue (Meth blue), azure A, and azure B for 2 hours. Data represent the mean and SEM of 4 independent experiments. Statistical differences were evaluated with one-way ANOVA (p ≤ 0.0001) and post hoc Bonferroni test comparing PBS or phenothiazine-treated cells with pCep4β (#) or pCep4βCasp6p20p10-transfected HCT116 cells (*). ###p < 0.001, *p < 0.05. (**b**) Western blot analysis of transfected- or non-transfected HCT116 cells shown in panel a for active Casp6p20 subunit (p20), full length Casp6 lacking its pro-domain; Casp6p20p10 (p20p10), Tubulin-cleaved by Casp6 (TubΔCasp6), full length Tubulin and β-actin. (**c**) Densitometric quantification of the levels of Tub∆Casp6 in three independent experiments as shown in panel b. Data represent the mean and SEM of 3 independent experiments. Statistical evaluation conducted as described in a. ###p < 0.001, **p < 0.01, ***p < 0.001. (**d**) Casp6 VEIDase enzymatic activity in serum-deprived primary human neuron cultures treated with 100 μM methylene blue, 50 μM azure A, and 10 μM azure B or PBS for 2 hours. Data represent the mean and SEM of 4 independent experiments. Statistical differences were conducted with a one-way ANOVA (p < 0.041) followed by a post hoc Bonferroni test comparing PBS or phenothiazine-treated cells with serum-treated (+serum; #) or serum-deprived PBS-treated cells (PBS; *) #p < 0.05, *p < 0.05, **p < 0.01). (**e**) Western blot analysis of neuronal protein extracts shown in panel d for levels of Tub∆Casp6 and full length tubulin. (**f**) Quantification of the levels of Tub∆Casp6/total tubulin shown in panel e. Data represent the mean and SEM of 3 independent experiments. Statistical evaluations by one-way ANOVA (p = 0.0315) followed by post hoc Dunnett’s test comparing to serum-treated (no significant difference) and serum deprived PBS-treated neurons (*p < 0.05). (**g**) Western blot analysis of the p20 active subunit of Casp6 (p20) and β-actin in proteins from serum-deprived human neurons.

To test the ability of phenothiazines to inhibit Casp6 in live cells, HCT116 cells, transfected with a self-activating form of Casp6 (Casp6p20p10) to induce high levels of Casp6 activity^22^ were treated for 2 hrs with 100 μM and 1 mM methylene blue, 50 μM azure A, and 10 μM and 100 μM azure B, concentrations determined to be non-toxic on non-transfected HCT116 cells. Methylene blue and azure B significantly inhibited Casp6 activity on the Ac-VEID-AFC peptide substrate (Fig. 4a). At 50 μM, azure A also showed a trend towards inhibition but it did not reach statistical significance. Western blot analyses revealed that while the levels of the cellular active p20 subunit of Casp6 were maintained, the tubulin cleaved by Casp6 (Tub∆Casp6) was significantly decreased in the cells treated with phenothiazines, thus clearly indicating functional inhibition of the cellular Casp6 activity (Fig. 4b,c).

To determine if these phenothiazines might inhibit caspases in the chief cell type affected in AD brains, human primary CNS neuron cultures were serum-deprived, a condition known to activate endogenously expressed Casp6^33^, and treated with phenothiazines. Higher concentrations of methylene blue and derivatives were more toxic to serum-treated human neuron cultures (Fig. 3d) than to the cell line so the lower concentrations were assessed. As previously observed, serum deprivation induced a 2-3 fold increase in Casp6 activity (Fig. 4d). Treatment of serum-deprived neurons for 2 hrs with 100 μM methylene blue, 50 μM azure A, or 10 μM azure B, significantly inhibited VEIDase activity *in vitro* (Fig. 4d), and Tub∆Casp6 in cells (Fig. 4e). Quantitation of 3 independent experiments indicated an increase in Tub∆Casp6/Tub in serum deprivation, but it did not reach statistical significance because of variability in human neurons (Fig. 4f). Nevertheless, methylene blue returned the levels to the basal levels observed in serum-treated neurons and azure A and azure B significantly decreased the levels compared to PBS-treated serum-deprived neurons. The levels of the active p20 Casp6 subunit remained the same with or without phenothiazine treatments, consistent with inhibition of the enzyme rather than increased turnover of active Casp6 (Fig. 4g). There is a slight discrepancy in the inhibition measured by VEIDase activity and that reported by the Tub∆Casp6/Tub level in the human neurons. The levels of Tub∆Casp6 are much lower in azure A and azure B-treated neurons compared to the methylene blue. This is likely due either an effect on the turnover of Tub∆Casp6, on the ability of the Tub∆Casp6 to bind to the caspase or to oxidative effects on the Tubulin. Nevertheless, together, these results demonstrate that phenothiazines can inhibit caspase activity in live cells, and importantly in human neurons.

### Methylene Blue and its derivatives inhibit the activity of Casp3 *in vivo*

**Figure 5:**
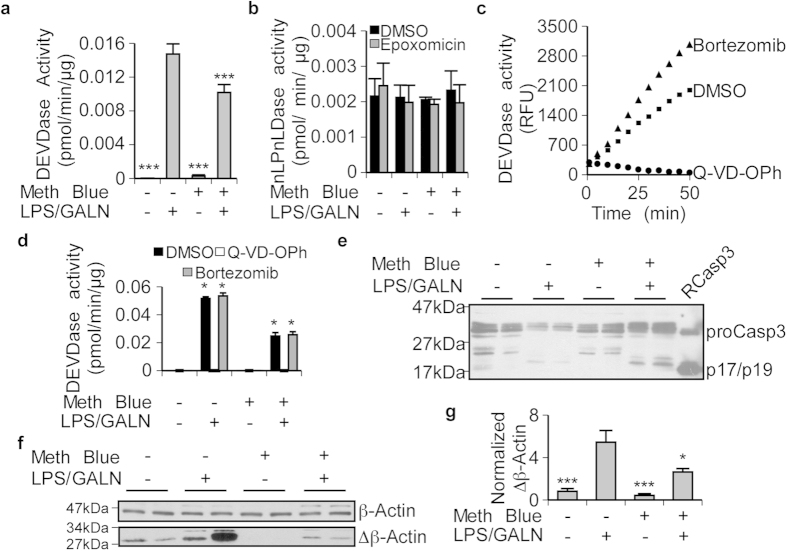
Methylene Blue inhibits Casp3 activity in liver of mice treated with LPS/GALN. (**a**) Casp3 activity was assessed in liver protein extracts from mice treated 3 days with methylene blue (3 mg/kg) or vehicle and then injected with LPS/GALN or PBS. Data represent the mean and SEM of 8 independent experiments. Statistical differences were assessed with one-way ANOVA (p < 0.0001) and a post hoc Dunnett’s test comparing all conditions to LPS/GALN-treated conditions ***p < 0.001. (**b**) Proteasomal nLPnLDase activity measured in liver protein extracts from mice treated with methylene blue and/or LPS/GALN in the presence or absence of 0.1 μM proteasome inhibitor epoxomicin. Data represent the mean and SEM of 3 independent experiments. No statistical differences were observed. (**c**) RCasp3 activity after treatment with DMSO, 1 μM of the pan-caspase inhibitor Q-VD-OPh, or 0.1 μM of the reversible proteasome inhibitor bortezomib. (**d**) DEVDase activity in liver protein extracts from mice treated with or without methylene blue and LPS/GALN after addition of DMSO, 1 μM Q-VD-OPh, or 0.1 μM bortezomib. Data represent the mean and SEM of 3 independent experiments. Statistical differences were assessed in a one-way ANOVA (p < 0.0001) followed with a post hoc Dunnett’s test comparing all conditions to DMSO in control-treated mice (*). DEVDase activity in DMSO treated protein extracts from LPS/GALN (no methylene blue)-treated mice did not change significantly in the presence of bortezomib. (**e**) Western blot of liver protein extracts from two different mice per group showing levels of proCasp3 and cleaved-Casp3 (p17/p19). (**f**) Western blot of liver protein extracts from two different mice per group showing levels of cleaved β actin and β-actin. (**g**) Quantitative analyses of cleaved β-actin over full-length actin levels in liver protein extracts from mice treated with methylene blue or vehicle in the presence or absence of LPS/GALN. Data represent the mean and SEM of 8 independent experiments. Statistical evaluations were conducted as described in a.

To determine if methylene blue has the ability to inhibit caspases *in vivo*, wild type male C57BL6/J mice were given 3 mg/kg of methylene blue by gavage and injected with lipopolysaccharides (LPS)/galactosamine (GALN), a well-known model to assess drugs against liver Casp3 activity^34^. As expected, while control- and methylene blue-treated mouse livers did not show significant Casp3 DEVDase activity, control mice injected with LPS/GALN showed strong liver Casp3 DEVDase activity (Fig. 5a). Mouse liver protein extracts from mice pre-treated with methylene blue and injected with LPS/GALN showed significantly less Casp3 activity (Fig. 5a).

Caspase substrates can be cleaved by the caspases-like activity of the proteasome. Therefore, we tested Ac-nLPnLD-AMC as a specific substrate for the caspase-like activity of the proteasome^35^. RCasp3 was unable to cleave Ac-nLPnLD-AMC, whereas human neuronal extracts did possess the expected nLPnLDase proteasomal activity (see supplementary Fig. S2 online). Treatment with LPS/GALN had no effect on nLPnLDase activity in mouse liver protein extracts in the presence or absence of the irreversible proteasome inhibitor epoxomicin (Fig. 5b), nor was Casp3 DEVDase activity affected by epoxomicin (see supplementary Fig. S2 online). In addition, the reversible proteasome inhibitor, bortezomib, did not inhibit RCasp3 activity *in vitro* (Fig. 5c) nor did it prevent DEVDase activity in LPS/GALN-treated mouse liver protein extracts (Fig. 5d). However, nLPnLDase proteasomal activity was significantly inhibited by bortezomib (see supplementary Fig. S2 online). Furthermore, the pan-caspase inhibitor Q-VD-OPh abrogated RCasp3 activity (Fig. 5c) and DEVDase activity in mouse liver protein extracts (Fig. 5d). Therefore, the DEVDase activity observed in the mice livers after LPS/GALN challenge is not due to the proteasome activity but to caspase activity. These results strongly support methylene blue inhibition of Casp3 *in vivo*.

The possibility that the lower Casp3 activity was the result of less Casp3 enzyme in the methylene blue-treated LPS/GALN mice livers was excluded since both the proCasp3 enzyme and its active p17/p19 subunits were elevated in the methylene blue/LPS/GALN treated mice compared to the other three treatments (Fig. 5e). Higher levels of enzyme may indicate that methylene blue delays turnover of the proCasp3 and its active subunits despite reducing the activity. Casp3 can also cleave β-actin^36^, therefore levels of cleaved β-actin were used to assess the functional inhibition of Casp3 *in vivo*. Consistent with the lower levels of caspase DEVDase activity, the levels of cleaved β-actin relative to total β-actin were significantly lower in methylene blue/LPS/GALN-treated mouse liver protein extracts compared to LPS/GALN treatment alone (Fig. 5f,g). These results show that methylene blue can inhibit active caspases *in vivo*.

### Methylene blue inhibits Casp6 by oxidation of catalytic cysteine Cys163

**Figure 6:**
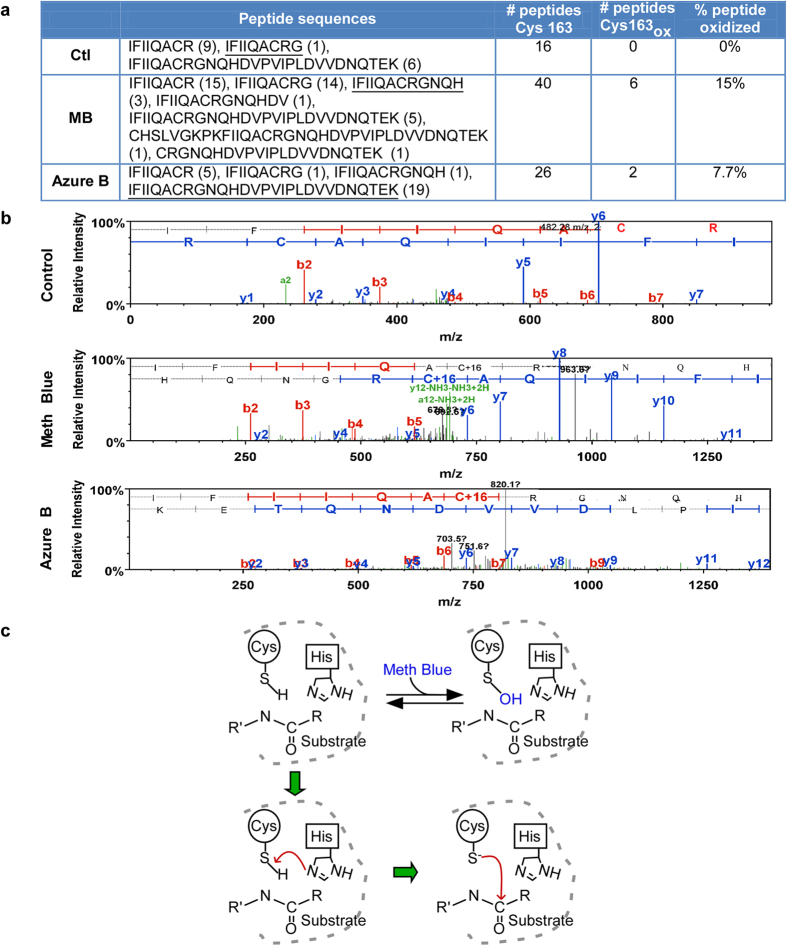
Methylene blue and azure B oxidizes the catalytic Cys163 of active Casp6. (**a**) The table represents the number of peptide sequences containing Cys163 obtained by MS/MS and the number of peptides showing Cys163 oxidation. The underlined peptide sequence spectrums are represented in panel b. The percentage of peptides oxidized represents the number of peptides containing an oxidized Cys163 divided by the total number of peptides containing the Cys163 in its sequence. (**b**) MS/MS spectra showing the oxidized (+16) catalytic Cys163 of Casp6 in the presence of methylene blue or azure B. (**c**) Schematic representation of Casp6 active site (top left panel). The catalytic mechanism of Casp6 is a multi-step process. The first step (bottom left panel) involves the de-protonation of the active site cysteine thiol by a histidine residue (His 121), thus activating the enzyme. The next step is a nucleophilic attack by the thiolate on the substrate’s peptide carbonyl carbon that subsequently leads to cleavage of the substrate (bottom right panel). However, in presence of methylene blue, the Cys163 thiol group is sulfenated and unable to attack the substrate (top left and right panels).

Mass spectrometric analyses of Casp6 showed the catalytic Cys163 of Casp6 to be oxidized into sulfenic acid (R-SOH) by both methylene blue and azure B (Fig. 6a,b). LC/MS/MS analyses did not reveal any peptide containing oxidized Cys163 in untreated recombinant Casp6, but 15% and 7.7% of sequenced peptides contained oxidized Cys163 in recombinant Casp6 treated with methylene blue and azure B, respectively (Fig. 6a). The levels of oxidation are consistent with the reversibility of sulfenation^37^. The sulfenation of Cys163 by methylene blue and azure B thus prevents the sulfur from attacking the scissile carbonyl of the peptide substrate, thereby rendering the caspase inactive (Fig. 6c).

## Discussion

Our study provides the identification of a post-translational regulatory modification of the caspase catalytic cysteine and adds a new group of cysteinyl enzymes that are regulated by sulfenation. Oxidation-dependent inhibition of caspases, mainly Casp3, has been demonstrated previously, but the chemical modification was not identified^38^,^39^,^40^. Our results add caspases to the list of 47 other proteins, including five other cysteinyl proteases, regulated post-translationally by sulfenation^37^. Sulfenation could transiently or locally regulate caspase activity, since all caspases are cysteinyl proteases. While cellular Casp3 is under strong regulation by the inhibitor of apoptosis proteins^41^, Casp1 and Casp6 do not have known cellular protein inhibitors, although Casp6 can be inhibited in an allosteric fashion by phosphorylation and zinc^42^,^43^.

The inhibition of Casp1, Casp3, and Casp6 could have a major influence on inflammation, apoptosis and neurodegeneration, respectively. Casp1 is the main producer of interleukin-1-beta, which can mediate several neuroinflammatory processes^44^, and is elevated early in AD brains^45^,^46^. Casp3 is an executioner caspase essential to apoptotic cell death and is also involved in neuronal function^47^. Casp6 is associated with axonal degeneration^26^,^29^. Oxidative stress increases in most cells of the body with aging and thus the oxidation of caspase catalytic cysteines could depress caspase-mediated functions *in vivo*. Similarly, inhibition of Casp1 and Casp6 by methylene blue could have beneficial effects by reducing inflammatory processes and axonal degeneration, but inhibition of Casp3 could alter neuronal function or promote cancer cell survival and be detrimental to tissue homeostasis. It is therefore important to monitor patients who are chronically treated with phenothiazines for deregulated inflammatory responses and cancers, especially in individuals at risk for these conditions.

Beneficial effects observed in AD clinical trials will have to consider the possibility that phenothiazines may benefit cognitive decline in AD not only by disaggregating Tau but by inhibiting Casp6-mediated axonal degeneration. The IC_50_ against Tau aggregation ranges from 2 μM 3 to 31 μM 1 for both methylene blue and azure B. The IC_50_ of methylene blue and azure B against Casp6 cleavage of tubulin are 10.6 μM and 34.4 μM, respectively, whereas azure A is 0.5 μM. Therefore, the abundant levels of active Casp6 observed in AD brains^48^ may be inactivated by phenothiazines used at clinical concentrations to promote Tau disaggregation.

Compared to other caspase inhibitors, which have IC_50_ in the nM or pM range, phenothiazines are weak inhibitors^49^, phenothiazines are weak inhibitors. However, the fact that phenothiazines are non-toxic at high concentrations in humans and can reach mean plasma levels of 5 μM, traverse the blood brain barrier, and are over 70% bioavailable after an oral ingestion^50^ indicate that concentrations capable of inhibiting caspases could be reached in the brain.

Methylene blue and its derivatives have other pleiotropic effects that could benefit AD (reviewed by^1^). Phenothiazines inhibit acetyl-cholinesterase and butyrylcholinesterase, thereby possibly increasing levels of acetylcholine^51^,^52^,^53^. Interestingly, reversible acetylcholinesterase inhibitors are the main drugs used against AD presently. Methylene blue also inhibits monoamine oxidase thereby increasing the levels of 5-hydroxytryptamine involved in several nervous system functions including mood and cognition^54^. Methylene blue enhances mitochondrial function^55^, which is impaired in AD brains, and reduces levels of amyloid beta peptide in transgenic mice models^56^. The inhibitory function of methylene blue against Casp6 activity may also benefit Huntington disease^57^, axonal degeneration of the nervous system^24^,^25^,^26^,^27^,^28^,^29^, or other conditions where Casp6 activity mediates pathogenesis. Thus, it will be important to dissect out the exact mechanism of methylene blue *in vivo* to fully understand how phenothiazines benefit age-dependent cognitive deficits and AD. Autopsied brains from AD individuals treated with phenothiazines will be invaluable to assess the effect of methylene blue on neurofibrillary tangles and Casp6 activity and obtain more selective therapies against AD.

These results imply that methylene blue or its derivatives could (1) have an additional positive effect against AD by inhibiting caspases, (2) be used as a drug to prevent caspase activation in other degenerative conditions, and (3) predispose chronically treated individuals to cancer via the inhibition of caspases. In conclusion, this study identifies an important mechanism of action of methylene blue against activity of caspases that could impact tissue homeostasis in pathological and age-dependent conditions.

## Methods

### DNA constructs

The pET23b (+) recombinant human proCasp6-His*tag was a kind gift from Dr Guy Salvesen (Sandford-Burnham Medical Research Institute, CA) and our laboratory cloned the human Casp6p20p10 in pCep4β vector^22^.

### Protein expression and purification

pET23b (+) recombinant pro-Casp6-His*tag was purified from *E. Coli* BL21 (DE3)pLysS (Stratagene, La Jolla, CA, USA)^58^. Protein concentration was determined by the Bradford protein assay (BioRad, Mississauga, ON, CA). Active Casp6 and Casp3 represented >99% of the purified enzyme, as verified by titration against their respective inhibitors^58^ (see supplementary Fig. S3 online). RCasp1 (0.15 units) (specific activity = 5000 U/mg) was added per 50 μL reaction yielding a 20 nM concentration.

### Caspase activity assays

Casp6 activity was assessed by *in vitro* fluorogenic assays with the Ac-Val-Glu-Ile-Asp-7-Amino-4-trifluoromethyl-couramin substrate (Ac-VEID-AFC: Enzo LifeSciences, NY, USA) in Stennicke’s buffer [SB: 20 mM piperazine-N, N-bis (2-ethanesulfonic acid (BioShop Canada Inc., Burlington, Ontario, CA) pH 7.2, 30 mM NaCl, 1 mM EDTA, 0.1% CHAPS, 10% sucrose]^59^. Briefly, the reaction mix consisted of 20 nM RCasp6 or 20–30 μg cellular protein extracts, 1 X SB, 10 μM DTT, and 10 μM Ac-VEID-AFC substrate. Methylene Blue (IUPAC: [7-(dimethylamino)phenothiazin-3-ylidene]-dimethylazanium;chloride, Sigma-Aldrich, St. Louis, MO, USA), azure A (IUPAC (7-aminophenothiazin-3-ylidene)-dimethylazanium;chloride) and azure B (dimethyl-[7-(methylamino)phenothiazin-3-ylidene]azanium;chloride,) from MP Biomedicals, Solon, OH, USA were added and the activity was measured in a black clear bottom 96-well plate at 37 °C in the Synergy H4 plate reader (BioTek, Winooski, VT, USA) every two minutes for 100 minutes. An AFC (Sigma-Aldrich) standard curve was used and the assay was gain adjusted to 12.5 μM AFC (ex: 380 nm, em: 505 nm). Activity assays for RCasp1 (Biovision, Millpitas, CA, USA) and RCasp3 (Enzo Lifesciences) used Z-Tyr-Val-Ala-Asp-7-Amino-4-trifluoromethylcoumarin (z-YVAD-AFC: Enzo LifeSciences,), and Ac-Asp-Glu-Val-Asp-7-Amino-4-trifluoromethylcouramin (Ac-DEVD-AFC: Enzo LifeSciences), respectively. To confirm that these substrates report caspase activity and not proteasomal caspase-like enzyme activity, 1 μM pan-caspase inhibitor, Q-VD-OPh (Sigma-Aldrich) or 0.1 μM proteasome inhibitor, bortezomib (Calbiochem, MA, USA) were tested on 20 nM RCasp3 or 40–60 μg of mouse liver protein extracts.

### IC_50_ determination using an *in vitro* tubulin cleavage assay

The IC_50_ for methylene blue, azure A, and azure B was determined by performing an *in vitro* tubulin cleavage assay because concentrations of phenothiazines above 100 μM interfered with both fluorescence (see supplementary Fig. S1 online) and luminescence (not shown) enzyme assays. Phenothiazines were mixed with 20 nM RCasp6 or RCasp3, 10 μM DTT, and 7.5 nM BSA in SB and 36 μg HCT116 protein extracts added before incubation at 37 °C for 10 hours, western blotting, and densitometry for tubulin-cleaved by Casp6 (Tub∆Casp6) and total tubulin as described in the western blot section. The IC_50_ was determined by a non-linear regression fit for log inhibitor vs caspase activity with a Hill slope of −1 using GraphPad Prism 5.0.

### Lineweaver-Burk Plot

Phenothiazines were added to 10 nM RCasp6 and enzyme activity was measured on nine different Ac-VEID-AFC concentrations (2.5 μM to 200 μM). To estimate K_i_ values, the apparent K_m_ and V_max_ values for the Ac-VEID-AFC substrate in the presence and absence of phenothiazines were determined via non-linear regression analysis of the corresponding Michaelis-Menten graphs (v vs. [S]). The K_i_ values and type of inhibition was subsequently estimated from the x-axis (K_m_ values) and y-axis (V_max_) intercepts of plots versus inhibitor concentrations. Linear and non-linear regression analyses were performed using the Prism version 5.0 software package.

### Cell culture treatments with methylene blue and derivatives

Human colon carcinoma (HCT116) cells, cultured as recommended (ATCC: Manassas, VA, USA), were transfected with 1 μg pCep4βCasp6p20p10 and 8 μg polyethyleneimine (Polysciences Inc., Warrington, PA, USA), and grown 24 hrs before treatment with PBS and phenothiazines for 2 hours. Human primary neurons were cultured from fetal brain cortical areas obtained under ethical guidelines and approved by the McGill University’s institutional review board as previously described^60^. Human primary neurons plated in poly-L-lysine -coated 6-well plates at a density of 3 × 10^6^ cells/mL were serum-deprived for 2 hours in the presence of phenothiazines or an equivalent volume of PBS. Proteins were harvested in cell lysis buffer (CLB; 50mM HEPES, 0.1% CHAPS, 0.1mM EDTA).

### Cell viability assays: Microscopic analysis

HCT116 cells, plated at a density of 1 × 10^5^ cells/well and human neurons, plated at a density of 6 × 10^6^ cells/well on poly-L-lysine were treated with PBS or phenothiazines for 2 hours. The media was replaced before acquiring images with the Nikon Eclipse Ti microscope and the NIS-Elements (Version 3.10) software. ***MTT assays***: Cells were plated in 96-well plates at 1 × 10^4^ or 1 × 10^5^ cells per well for 24 hours before treatments with phenothiazines or PBS. After 2 hours, fresh media containing 0.5 μg/ml MTT (3-(4,5-dimethylthiazol-2-yl)-2,5-diphenyltetrazolium bromide) (Sigma-Aldrich) was added and the cells were incubated for 4 hours at 37 °C in 5% CO_2_. The media was removed, formazan crystals were dissolved in DMSO, and the absorbance was measured at 560 nm and 670 nm using the BioTek Synergy H4 plate reader.

### Proteasome assay

The caspase-like activity of the proteasome was measured using 10 μM Ac-Nle-Pro-Nle-Asp-AMC (Ac-nLPnLD-AMC, Enzo Lifesciences) as described for caspase activity assays except for the use of 10 mM DTT and a 7-amino-4-methylcoumarin (AMC, Sigma-Aldrich) standard curves. Mouse liver protein extracts were treated with 0.1 μM of epoxomicin (Enzo Lifesciences), bortezomib, 1 μM of Q-VD-OPh, or equal volumes of DMSO for 20 mins on ice before measuring proteasome or caspase activities.

### Western blot analyses

The 10630 (1:10,000) and GN60622 (1:10,000) neoepitope antibodies against the p20 subunit of active Casp6^16^ and Tub∆Casp6^23^, respectively, were generated in our laboratory. The β-actin clone AC-15 (1:5000, Sigma-Aldrich), Casp6 p10 clone B93-4 (1:250, BD Canada, Mississauga, ON, CA), Casp3, and full-length α-tubulin (1:1000, Cell Signaling Technology Inc., Danvers, MA, USA) antibodies were diluted in 5% non-fat dry milk in Tris-buffered saline containing 0.1% Tween-20 (Sigma-Aldrich). Secondary anti-mouse (1:5000, GE Healthcare Life Sciences, Baie D’Urfe, QC, CA) and anti-rabbit antibodies (1:5000, Dako, Burlington, ON, CA) conjugated to horseradish peroxidase (HRP) were used to detect immunoreactive proteins using ECL prime western blotting detection reagent (GE Healthcare Life Sciences) and Kodak BioMax MR film (Kodak, Rochester, NY, USA). Secondary anti-mouse conjugated to alkaline phosphatase (Jackson Immunoresearch Laboratories Inc., West Grove, PA) was developed with nitro-blue tetrazolium (ThermoSci) and 5-bromo-4-chloro-3′-indolyphosphate (ThermoSci). The western blots were scanned with an HP scanner and densitometry was performed using ImageJ software (NIH, Bethesda, MD) by rendering tiff images into an 8-bit format and measuring the intensity values above background. Images were not modified except to adjust the contrast for the entire blot.

### LPS/galactosamine treatment of C57BL6/J mice

All animal procedures followed the Canadian Council on Animal Care guidelines and were approved by the McGill Animal care committees. Male C57BL6/J mice (10–12 weeks old; Jackson Laboratories (Bar Harbor, ME, USA) were gavaged with 3 mg/kg dose of methylene blue or vehicle (0.5% methylcellulose (Sigma)) for 3 consecutive days. On the 3^rd^ day, after gavage, a mixture of 100 mg/kg of LPS (Sigma) and 700 mg/kg of D-(+)-galactosamine (GALN) (Sigma) or PBS was injected i.p.^34^. The mice were sacrificed using isofluorane/CO_2_ 5.5 hours after the i.p. injection. Left lobe liver sections (50–150 mg) were frozen rapidly and ground glass homogenized with 10x volume of iced CLB supplemented with fresh protease inhibitors (38 μg/mL AEBSF, 0.5 μg/mL leupeptin, 0.1 μg/mL TLCK, 0.1 μg/mL pepstatin; Sigma). The homogenates were centrifuged at 1000 × g at 4 °C and the supernatant analysed for caspase activity in a blind fashion.

### Electrospray ionization mass spectroscopy

RCasp6 (1 mg/ml) in 50 mM Tris, pH 8.0, 100 mM NaCl, 1 mM DTT was incubated for 15 min at 37 °C, divided equally in three tubes, and 2 mM methylene blue, 2 mM azure B or buffer were added to each tube and incubated for 45 min at 37 °C. The samples were snap-frozen in dry ice and ethanol, and stored at −80 °C until analysis at the IRIC Mass Spectrometry core facility (U. de Montreal, Montreal, Quebec, Canada). Briefly, 10 μg RCasp6 was digested with 1 μg of trypsin for 8 h at 37 °C, desalted with Ziptips, loaded on a homemade C18 pre-column (0.3 mm i.d. × 5 mm) connected directly to the switching valve and separated on a homemade reversed-phase column (150 μm i.d. × 150 mm) with a 56-min gradient from 10–60% acetonitrile (0.2% FA) and a 600 nl/min flow rate on a NanoLC-2D system (Eksigent) connected to an LTQ-Orbitrap Elite (Thermo Fisher Scientific). Each MS spectrum acquired with a 60,000 resolution was followed by 12 MS/MS spectra, where the 12 most abundant multiply charged ions were selected for MS/MS sequencing. Tandem MS experiments were performed using collision-induced dissociation in the linear ion trap. Peaks were identified using Mascot version 2.4 (Matrix science) and peptide sequence were blasted against the Human Uniprot database (301,754 sequences). Tolerance was set at 15 ppm for precursor and 0.5 Da for fragment ions during data processing. The occurrence of cysteine oxidations was considered.

### Statistical Analyses

The number of independent experiments and statistical analyses conducted with ANOVA and post hoc Bonferroni or Dunnett’s tests were indicated in the figure legends.

## Additional Information

**How to cite this article**: Pakavathkumar, P. *et al.* Methylene Blue Inhibits Caspases by Oxidation of the Catalytic Cysteine. *Sci. Rep.* **5**, 13730; doi: 10.1038/srep13730 (2015).

## Supplementary Material

Supplementary Information
