# Supplementary material for: Methylene Blue Inhibits Caspases by Oxidation of the Catalytic Cysteine
Source: Sci Rep. 2015 Sep 24;5:13730. doi: 10.1038/srep13730 (PMC4585840; doi:10.1038/srep13730)
Supplement: Supplementary Information [file srep13730-s1.pdf]

Methylene Blue Inhibits Caspases by Oxidation of the Catalytic Cysteine.

Prateep Pakavathkumar[1,2], Gyanesh Sharma[1,2], Vikas Kaushal[1,2], Bénédicte Foveau[1,2], Andrea C. LeBlanc[1,2*]

Prateep Pakavathkumar and Gyanesh Sharma have contributed equally to this manuscript.

[1] Bloomfield Center for Research in Aging, Lady Davis Institute for Medical Research, Jewish General Hospital, Montreal, Quebec, Canada

[2] Department of Neurology and Neurosurgery, McGill University, Montreal, Quebec, Canada

To whom correspondence should be addressed: Andrea LeBlanc, PhD, Bloomfield Center for Research in Aging, Lady Davis Institute for Medical Research, Sir Mortimer B Davis Jewish General Hospital, 3755 ch. Côte Ste-Catherine, Montréal, QC, Canada H3T 1E2. Tel.: +1 (514) 340 8222 ext 4976. Fax.: +1 (514) 340 8295. e-mail address: andrea.leblanc@mcgill.ca

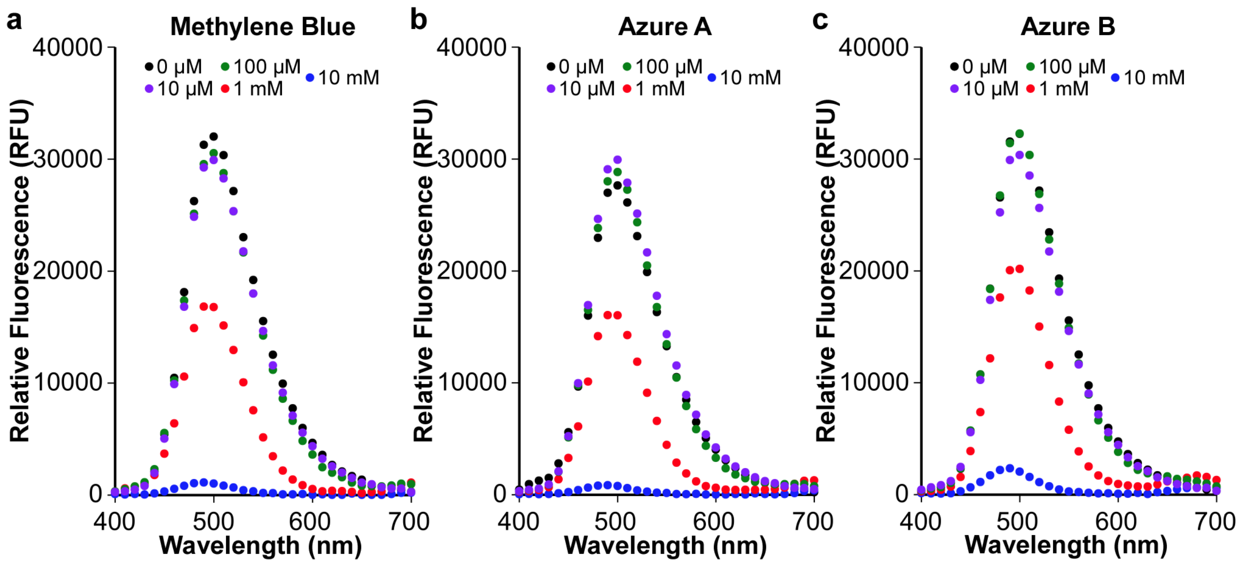

Supplementary Figure S1. **Intrinsic fluorescence of phenothiazines at different concentrations.** (**a**) Fluorescence emission spectrum of AFC in presence of 0, 10 µM, 100 µM, 1mM, 10 mM of methylene Blue, (**b**) azure A, and (**c**) azure B, respectively.

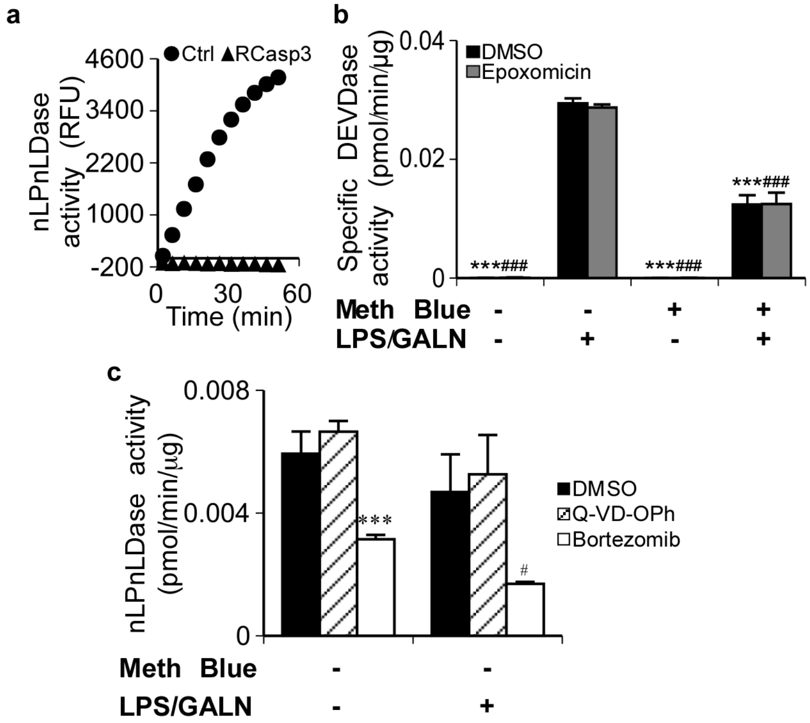

Supplementary Figure S2. Proteasome inhibitors do not inhibit DEVDase activity in mice liver extracts. (**a**) Proteasomal caspase-like activity measured by fluorescence of cleaved Ac-nLPnLD-AMC substrate in human neuronal extracts (Ctrl) and by recombinant active Caspase-3 (RCasp3). (**b**) Casp3 DEVDase activity measured in liver protein extracts from mice treated with methylene blue and/or LPS/GALN in the presence or absence of 0.1 µM of the irreversible proteasome inhibitor, epoxomicin. (**c**) nLPnLDase activity in liver protein extracts from mice treated with or without methylene blue and LPS/GALN after addition of DMSO, 1 µM Q-VD-OPh pan caspase inhibitor, or 0.1 µM bortezomib proteasome inhibitor.

Supplementary Figure. S3, Pakavathkumar et al., Methylene blue inhibition of caspases

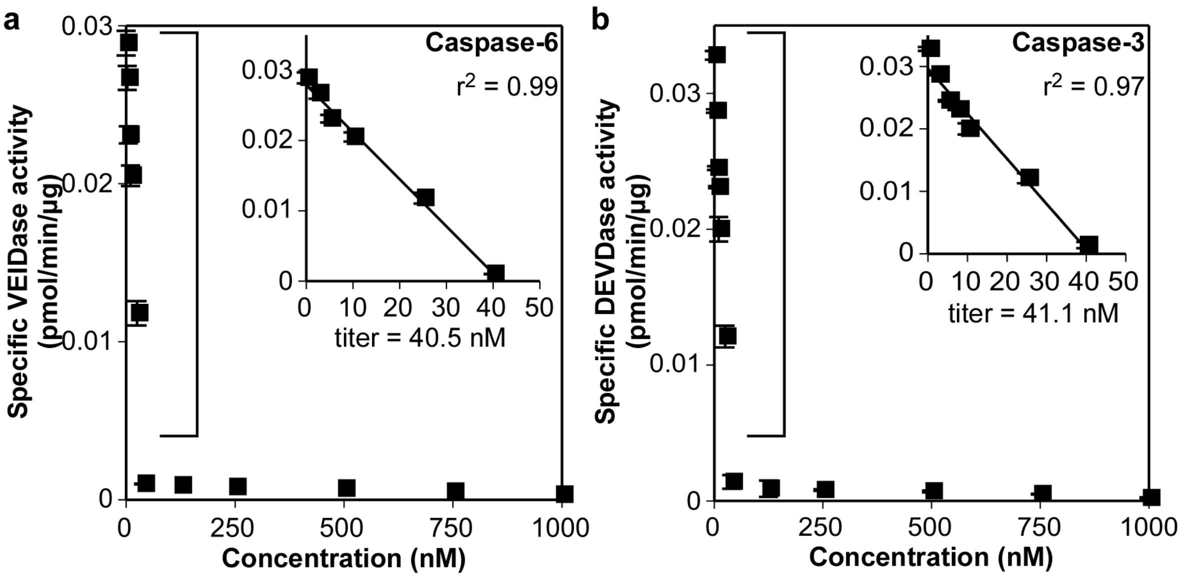

Supplementary Figure S3. **Recombinant Caspases are highly active and functional**. (**a&b**) The active-enzyme concentration for recombinant Caspase-6 and recombinant Caspase-3 was calculated by titrating the enzymes with the irreversible inhibitor, Z-VAD-fmk, using Ac-VEID-AFC and Ac-DEVD-AFC as substrates, respectively. The bracket in panel a and b indicate the values used to obtain the active recombinant Caspase-6 and recombinant Caspase-3 concentration by linear regression (inset). The x-axis intercept (titer) represents the concentration of the active enzyme in the assay. Data represent the mean ± S.D. of three independent experiments.

METHODS

Active Enzyme Measurements: Active RCasp6 and RCasp3 concentration was measured as previously described in (1). Briefly, it is a two-step assay. First the enzyme (final concentration 20 nM) is incubated with the titrant in concentrations ranging from 0 to 4 times the estimated caspase concentration in a low volume (25 µL) in assay buffer for 15 min at 37 °C. The titrant of choice is the irreversible caspase inhibitor benzoxycarbonyl-Val-Ala-Asp-fluoromethylketone (z-VAD-FMK) (Enzo LifeSciences, NY, USA). After the enzyme-titrant equilibrium is reached, the assay is diluted by the addition of excess substrate, VEID-AFC and DEVD-AFC and the remaining activity is determine with a simple enzymatic assay. The concentration of the active enzyme is determined by plotting the relative hydrolysis rate against the concentration of the titrant. The x-axis intercept of the tangent to the slope at low titrant concentration gives the active caspase concentration in the assay.

***Fluorescence Quenching**:* AFC (12.5 µM) in Stennicke buffer was mixed with varying concentrations of methylene blue, Azure A and Azure B (10 mM, 1 mM, 0.1 mM, and 100 µM). A fluorescence emission spectrum was generated between 400 to 700 by exciting at 380 nm for each phenothiazine compound. The AFC fluorescence was gain-adjusted to 45000 relative fluorescence units.

Caspase activity assays: Casp6 activity was assessed by *in vitro* fluorogenic assays using Ac-Val-Glu-Ile-Asp-7-Amino-4-trifluoromethyl-couramin) (Ac-VEID-AFC: Enzo LifeSciences, NY, USA) as the Casp6 substrate. The activity was measured using Stennicke's buffer (SB) (20 mM piperazine-N, N-bis (2-ethanesulfonic acid) (PIPES: BioShop Canada Inc, Burlington, Ontario, CA) pH 7.2, 30 mM NaCl, 1 mM EDTA, 0.1% CHAPS, 10% sucrose) (2). Briefly, the reaction mix consisted of 20 nM recombinant Casp6, 1 X SB, 10 µM DTT, 10 µM VEID-AFC substrate and deionized water. Methylene Blue, azure A, and azure B were added in increasing concentrations (0-100 µM) to the reaction mix and the activity was measured in a black clear bottom 96-well plate (Costar, Corning, NY, USA) at 50 µL/well in duplicate at 37 °C in the Synergy H4 plate reader (BioTek) at excitation 380 nm and emission 505 nm every two minutes for 100 minutes. Fluorescence units were converted to the amounts of moles of AFC released based on standard curve of 0 - 12.5 µM free AFC. Cleavage rates were calculated from the linear phase of the assay. The activity is considered on a percentage scale where no inhibitor present is

equated at 100% activity of the enzyme. Activity assays for recombinant Casp3 was done similarly to recombinant Casp6, except the substrate used was Ac-Asp-Glu-Val-Asp-7-Amino-4-trifluoromethylcouramin (Ac-DEVD-AFC: Enzo Life Sciences, NY, USA).

Proteasome assay: The caspase-like activity of the proteasome was measured using the substrate Ac-Nle-Pro-Nle-Asp-AMC (Enzo LifeSciences, NY, USA) at a final concentration of 10 μM in Stennicke's buffer in the presence of 10 mM DTT. The assay was setup similarly to the caspase activity assay except for the use of a 7-amino-4-methylcoumarin (AMC, Sigma-Aldrich, St. Louis, MO, USA) standard curve. Mouse extracts were incubated with the irreversible proteasome inhibitor, 0.1 μM epoxomicin (Enzo LifeSciences, NY, USA), or 0.1 μM bortezomib (Calbiochem, MA, USA), 1 μM the pan-caspase inhibitor Q-VD-OPh (Sigma-Aldrich, St. Louis, MO, USA) or equal volumes of DMSO for 20 mins on ice before starting the assay. Recombinant Casp3 (20 nM) was used to test the specificity of the proteasomal substrate.

Statistical Analysis: For Caspase-3 activity assays, data represent the mean ± SEM of 3 livers from 3 animals and was analyzed using a one-way ANOVA with Dunnett's post hoc test (*** $p < 0.001$ in DMSO, and ### $p < 0.001$ in bortezomib) comparing all samples to LPS/GALN-treated mice in the absence of methylene blue, while proteasome assays were analyzed using Dunnett's post hoc test (*** $p < 0.001$ for mice not-treated with methylene blue or LPS, and # for mice treated with LPS only) compared all samples to DMSO.

SUPPLEMENTARY REFERENCE LIST

1. Denault, J., and Salvesen, G. (2002) Expression, Purification, and Characterization of Caspases. in *Current Protocols in Protein Science*. pp 21.13.21-21.13.15
2. Stennicke, H. R., and Salvesen, G. S. (1997) Biochemical characteristics of caspases-3, -6, -7, and -8. *The Journal of biological chemistry* **272**, 25719-25723.
